# In Vitro Effect of Elevated Ammonia and Urea Levels on Post-Thawed Bull Semen Sperm Characteristics

**DOI:** 10.3390/vetsci12100997

**Published:** 2025-10-15

**Authors:** Amine Abdelli, Mohamed Besbaci, Ziyad Al-Kass, Mokrane Iguer-Ouada, Jane M. Morrell

**Affiliations:** 1 Department of Agricultural Sciences, Laboratory of Management and Valorization of Natural Resources and Quality Assurance (LGVRNAQ), University of Bouira, Bouira 10000, Algeria; a.abdelli@univ-bouira.dz; 2 Laboratory of Biotechnology in Animal Reproduction, Institute of Veterinary Medicine, University of Blida1, Blida 09000, Algeria; besbaci@univ-blida.dz; 3 Department of Surgery and Theriogenology, College of Veterinary Medicine, University of Mosul, Mosul 41003, Iraq; ziyad.al.kass@slu.se; 4 Faculty of Nature and Life Sciences, Associated Laboratory in Marine Ecosystems and Aquaculture, University of Bejaia, Bejaia 06000, Algeria; imokrane@gmail.com; 5 Division of Reproduction, Department of Clinical Sciences, Swedish University of Agricultural Sciences, 750 07 Uppsala, Sweden

**Keywords:** bovine, co-incubation, urea, ammonia, semen characteristics

## Abstract

Elevated ammonia and urea, common byproducts of nitrogen metabolism, are increasingly found in dairy cows and may negatively impact reproductive function. However, their effects on bull sperm remain unclear. This study aimed to assess the impact of ammonia and urea at physiologically relevant concentrations on key sperm characteristics. Thawed bull semen was incubated under five treatment conditions: control (no added ammonia or urea), low urea (LU), high urea (HU), low ammonia (LA), and high ammonia (HA). Sperm motility and kinematics were assessed using Computer-Assisted Semen Analysis (CASA), and sperm viability, DNA integrity, mitochondrial membrane potential (MMP), and oxidative stress markers were evaluated using flow cytometry. HA significantly reduced MMP (*p* = 0.008) and several motility parameters, including progressive motility and velocity (VCL, VSL, ALH), compared to LA and control groups. A decrease in sperm viability was observed in the HA group compared to LA at the beginning of incubation. While HU reduced MMP (*p* = 0.002), sperm motility was not significantly affected compared to LU. No significant differences were found in DNA fragmentation or oxidative stress biomarkers between groups. These results highlight the impact of ammonia, specifically, on sperm mitochondrial function and motility, which are crucial for successful fertilization.

## Simple Summary

This study investigated the effects of ammonia and urea, at levels found in cattle fed high-nitrogen diets, on bull sperm quality. Thawed semen was incubated with low and high concentrations of each compound, and sperm motility, viability, DNA integrity, mitochondrial function, and oxidative stress were measured. High ammonia significantly reduced mitochondrial activity and sperm movement. High urea also decreased mitochondrial activity, but had less impact on motility. Ammonia reduced sperm viability at one hour. Neither compound affected DNA integrity or oxidative stress. These results suggest that ammonia, in particular, negatively impacts sperm function, which is crucial for fertilization.

## 1. Introduction

In recent decades, feeding practices for dairy cows have evolved significantly, with a strong focus on optimizing milk production. Attempts have been made to enhance milk yield, including feeding high-protein diets, with the inclusion of high rumen-degradable protein (RDP), aimed at maximizing protein synthesis and, consequently, milk production [1]. While high dietary nitrogen levels boost milk production, they may be linked to high urea nitrogen in blood and milk (PUN and MUN, respectively), which are thought to have adverse effects on fertility [2]. Previously, high nitrogen concentrations in dairy cows were observed to be associated with decreased fertility [3,4,5,6]. However, the evidence remains somewhat equivocal, and high dietary protein is likely to have a multifactorial effect on fertility. One proposed mechanism is the harmful effect of elevated levels of metabolites resulting from excess dietary protein, which can interfere with one or more stages of conception [7]. Therefore, feeding cows more protein than they require has been linked to increased nitrogenous metabolic byproducts (e.g., NH_3_ and urea) in blood and milk, which are used as markers of reduced fertility [6,8].

A negative association between elevated NH_3_ and urea concentrations and conception rates has been observed in dairy cows, particularly around insemination [5,7,9]. Some researchers have attributed conception failure to changes in uterine pH caused by increased NH_3_ levels, while others have linked it to the potential direct toxicity of urea and NH_3_ [7]. Both urea and NH_3_, which are end-products of protein metabolism, can diffuse into various body tissues, including the reproductive organs [4]. Moreover, a strong correlation has been observed between PUN concentrations, NH_3_ concentrations, and urea nitrogen levels in reproductive fluids, including oviductal–uterine fluid [10], and vaginal mucus [11] in dairy cows. Additionally, high concentrations of urea and NH_3_ have been shown to adversely affect male reproductive function and sperm quality across several species, including humans and livestock such as cattle [12,13]. Further underscoring this impact, an in vitro study showed that urea and ammonia together hindered ram spermatozoa penetration of synthetic mucus [14].

The importance of the oviduct–uterine environment for key aspects of fertilization, such as gamete transport, oocyte maturation, subsequent embryonic development and transport, is well established [15]. Changes in protein metabolism and the oviductal–uterine fluid environment can disrupt some of these crucial steps, thus impeding reproductive efficiency [16]. Several studies have investigated the impact of elevated urea and NH_3_ concentrations on oocyte maturation [15,17], fertilization [16,17], and embryo development [18,19]. However, to our knowledge, little research has focused on the impact of these nitrogenous breakdown products on sperm characteristics within the oviductal–uterine lumen following insemination. This study investigated the in vitro effects of elevated ammonia and urea concentrations on post-thawed bull semen characteristics.

## 2. Materials and Methods

### 2.1. Semen Source and Preparation

Frozen semen from a 4-year-old Swedish Red bull (Viking Genetics, Skara, Sweden), in good health and of proven fertility, was used. Semen collection and freezing took place according to standard protocols at the bull station [20]. For each replicate, ten straws from the same ejaculate were thawed in a 37 °C water bath for 20 s, then pooled and thoroughly mixed to ensure sample homogeneity. Sperm concentration was measured using a NucleoCounter^®^ SP 100™ (ChemoMetec, Allerød, Denmark) following established procedures [21].

### 2.2. Experimental Treatments and Incubation

After determining the sperm concentration, the pooled sample was divided into five aliquots and diluted to a standardized concentration of 2 × 10^6^ sperm/mL, each using one of five different extenders: (1) a control group (Control; basic TALP extender) and four treatment groups using TALP supplemented with either (2) high urea (HU; 26.9 mg/dL), (3) low urea (LU; 20.4 mg/dL), (4) high ammonia (HA; 1141 µmol/L ammonium chloride), or (5) low ammonia (LA; 1025 µmol/L ammonium chloride). These concentrations were selected to mimic physiological levels found in bovine uterine fluid on day 0 of the estrous cycle, reflecting varying plasma urea nitrogen [10]. The TALP medium was prepared according to a published protocol and contained 1 mg/mL sodium pyruvate, 3.1 mM KCl, 25 mM NaHCO_3_, 100 mM NaCl, 10 mM HEPES, 21.6 mM sodium lactate, 0.3 mM NaH_2_PO_4_, and 10 μg/mL penicillin and streptomycin. Sperm samples were incubated at 37 °C in 5% CO_2_ for 180 min. The experiment was performed in triplicate to ensure reproducibility.

Sperm motility was assessed at 0, 1, 2, and 4 h of incubation. Sperm motility and kinematics were assessed using a computer-assisted sperm analysis (CASA) system (SpermVision™ v. 3.5, Minitüb GmbH, Tiefenbach, Germany) mounted on an Olympus BX51 microscope. Observations were made under a 100× magnification (10× objective lens and 10× ocular) with a heated stage maintained at 38 °C. Image sequences were recorded at 60 frames per second, with 30 frames captured per field, corresponding to a trajectory duration of 0.5 s. A minimum of five fields were recorded per sample, and at least 500 sperm cells were analyzed per time point. CASA settings, including thresholds for motility classification and velocity parameters, were standardized across all analyses to ensure consistency and comparability. The following parameters were recorded: total motility (%, Mot), progressive motility (%, Prog Mot), curvilinear velocity (VCL, µm/s), straight-line velocity (VSL, µm/s), average path velocity (VAP, µm/s), linearity (LIN, %), straightness (STR, %), wobble (WOB, %), amplitude of lateral head displacement (ALH, µm), and beat-cross frequency (BCF, Hz).

Flow cytometric analyses were performed at 30 min, 1 h, and 2 h post-thaw (due to the 30 min preparation time required for these assays).

All chemicals were sourced from Sigma-Aldrich, Louis, MO, USA.

### 2.3. pH Measurement

The pH of each prepared extender solution (Control, HU, LU, HA, and LA) was measured immediately before each experimental replicate using a calibrated pH meter (PHM210, MeterLab^®^, Danderyd, Sweden), using pH 4 and 7 buffer solutions (VWR^®^ AVS TITRINORM, VWR International GmbH, Darmstadt, Germany).

### 2.4. Sperm Cell Characteristic Analysis

Sperm characteristics were evaluated using flow cytometry (FACSVerse, BDBiosciences; Franklin Lakes, NJ, USA).

#### 2.4.1. Viability

Sperm viability (membrane integrity) was analyzed using the Live/Dead^®^ Sperm Viability Kit (Invitrogen, L-7011, Waltham, MA, USA) following a published protocol for thawed bull semen [22]. Samples were stained with SYBR-14 (sperm with intact membranes) and propidium iodide (PI) (sperm with damaged membranes) and incubated for 10 min before flow cytometric analysis (30,000 sperm events).

#### 2.4.2. Reactive Oxygen Species (ROS)

Sperm subpopulations producing superoxide (O_2_^−^) and hydrogen peroxide (H_2_O_2_) were quantified using flow cytometry as described previously [22]. Sperm suspensions were stained with Hoechst 33258, hydroethidine (HE), and 2′,7′-dichlorodihydrofluorescein diacetate (H_2_DCFDA) and incubated in the dark for 30 min before analysis.

#### 2.4.3. Mitochondrial Membrane Potential (MMP)

MMP was assessed following staining with JC-1 for 30 min, and fluorescence was measured by flow cytometry (50,000 sperm events). Sperm were classified as having high or low MMP based on JC-1 fluorescence.

#### 2.4.4. Sperm Chromatin Structure Assay

DNA fragmentation was evaluated using a flow cytometric assay [23]. The sperm samples in TNE buffer were thawed on ice, subjected to partial DNA denaturation using Triton X-100, and stained with acridine orange (AO). Fluorescence was measured by flow cytometry (10,000 sperm events), and the proportion of sperm with red fluorescence (reflecting single strand DNA breaks) was calculated to provide the DNA fragmentation index (%DFI).

### 2.5. Statistical Analysis

Statistical analyses were performed in R (version 4.3.1) and RStudio (version 2024.04.1). Each treatment was performed in triplicate, and sperm characteristics were measured repeatedly at four incubation time points (0, 1, 2, and 4 h). Normality of residuals was assessed using Q–Q plots, and logarithmic transformation was applied when necessary. Parameters meeting normality assumptions were analyzed using repeated-measures ANOVA, with Tukey’s HSD applied for post hoc between-group comparisons. The Kruskal–Wallis test was used to analyze differences in pH. Figures were produced in GraphPad Prism (version 6.07).

## 3. Results

### 3.1. pH of the Prepared Incubation Media

The mean pH values of the prepared media were 7.80 ± 0.07 (HU), 7.73 ± 0.01 (LU), 7.82 ± 0.1 (HA), 7.78 ± 0.06 (LA), and 7.60 ± 0.14 (control), with no significant differences between groups (Table 1).

### 3.2. Sperm Motility Variables

#### 3.2.1. Incubation with Ammonia

Incubation with ammonia significantly affected sperm motility and kinematic parameters (Table 2). HA reduced total motility compared with LA (*p* = 0.02) and progressively reduced progressive motility compared with both control and LA groups (*p* < 0.05). These effects were evident at time 0 and remained significant through 2 h. By 4 h, WOB was also significantly reduced in the HA group compared with control (*p* < 0.05).

Kinematic descriptors were likewise impaired by ammonia. At 2 h, VAP, VCL, VSL, ALH, and BCF were all significantly lower in HA compared with control (*p* < 0.05). In particular, VAP was markedly reduced in HA compared with both LA and control (*p* = 0.01). Significant temporal effects were observed for VAP, VCL, VSL, ALH, and BCF (*p* < 0.05), and group × time interactions were detected for progressive motility, VAP, VCL, VSL, and ALH (*p* < 0.05).

#### 3.2.2. Incubation with Urea

The addition of 20.4 mg/dL and 26.9 mg/dL of urea to thawed bull sperm did not affect total or progressive motility, or any kinematic parameters. However, there was an observed effect of urea concentration (high or low) on progressive sperm motility, VAP, VCL, and ALH at 1 h and 2 h, and on VSL and BCF specifically at 1 h and 2 h, respectively. Over time, only VAP, VCL, VSL, and ALH showed significant changes. No significant group-by-time interactions were observed for any of the parameters (Table 3).

### 3.3. Flow Cytometric Analysis

#### 3.3.1. Incubation with Ammonia

**Figure 1:**
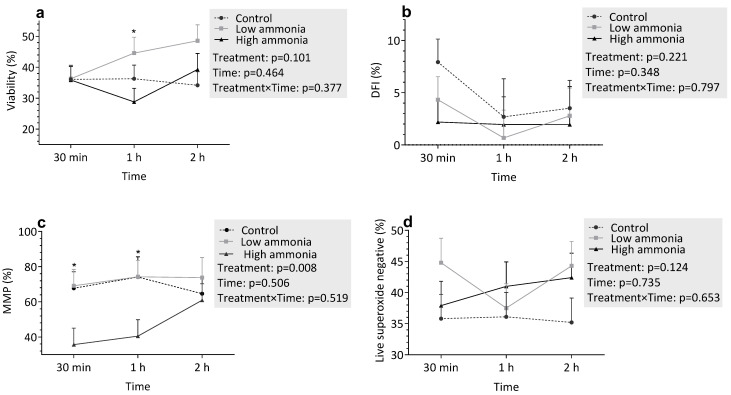

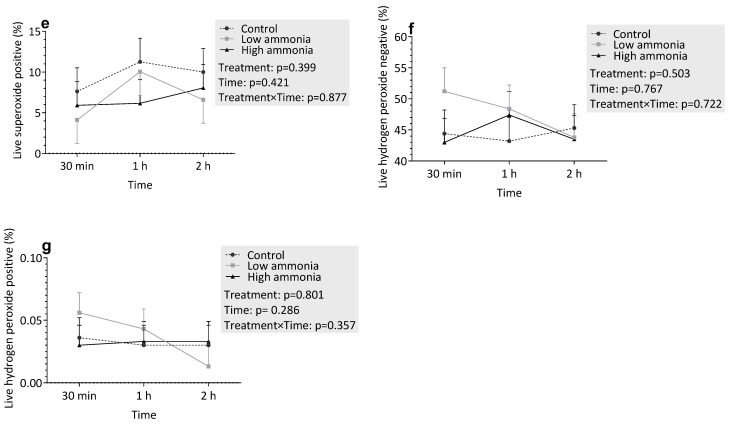
Flow cytometric evaluation of sperm parameters under varying ammonia treatments: (**a**) Viability, (**b**) DNA fragmentation index (DFI, %), (**c**) Sperm with high mitochondrial membrane potential (MMP,%), (**d**) Live superoxide-negative sperm, (**e**) Live superoxide-positive sperm, (**f**) Live hydrogen peroxide-negative sperm, (**g**) Live hydrogen peroxide-positive sperm. Spermatozoa were incubated in control (C), low ammonia (LA), and high ammonia (HA) treatments for 30 min, 1 h, and 2 h. Data represent least-squares means (LSMEANS) ± standard error (SE). An asterisk (*) above a time point indicates a statistically significant difference between groups (*p* < 0.05).

The effects of ammonia on various flow cytometric traits are shown in Figure 1. Ammonia treatment did not significantly DNA fragmentation (Figure 1b; *p* = 0.221). However, a statistically significant reduction in MMP was observed (Figure 1c; *p* = 0.002). Specifically, the HA group displayed consistently lower MMP values compared to the control and HA groups at 30 min and 1 h. HA concentrations significantly reduced viability at 1 h of incubation compared to LA concentrations. However, at the other time points, viability was similar across treatments.

Regarding oxidative stress markers, there were trends toward increased superoxide (Figure 1d,e; *p* > 0.05) and hydrogen peroxide (Figure 1f,g; *p* > 0.05) production with increasing ammonia concentrations. Over time, there were no significant changes in any of the measured parameters (*p* > 0.05) Similarly, the interaction between treatment and time was not significant for any parameter (*p* > 0.05).

#### 3.3.2. Incubation with Urea

**Figure 2:**
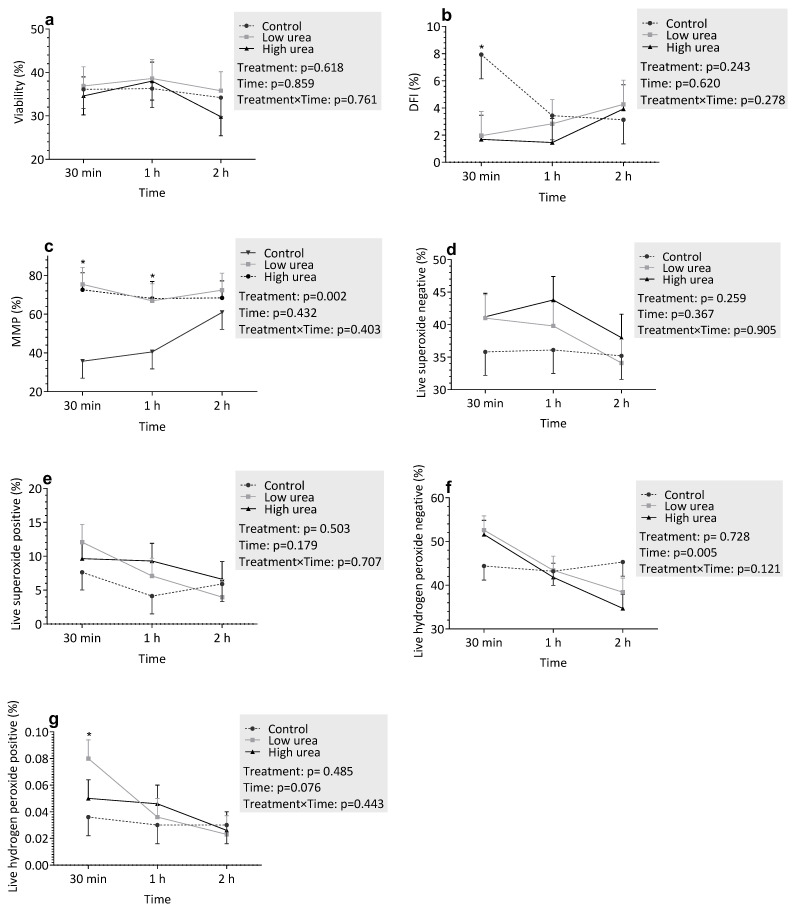
Flow cytometric evaluation of sperm parameters under varying urea treatments: (**a**) Viability, (**b**) DNA fragmentation index (DFI,%), (**c**) Sperm with high mitochondrial membrane potential (MMP,%), (**d**) Live superoxide-negative sperm, (**e**) Live superoxide-positive sperm, (**f**) Live hydrogen peroxide-negative sperm, (**g**) Live hydrogen peroxide-positive sperm. Spermatozoa were incubated in control (C), low ammonia (LA), and high ammonia (HA) treatments for 30 min, 1 h, and 2 h. Data represent least-squares means (LSMEANS) ± standard error (SE). An asterisk (*) above a time point indicates a statistically significant difference between groups (*p* < 0.05).

The effects of urea on various flow cytometric traits are shown in Figure 2. Urea treatment did not affect sperm viability (Figure 2a; *p* = 0.618). However, a significant reduction in MMP was observed (Figure 2c; *p* = 0.008), with HU groups displaying consistently lower values compared to the control and urea-low groups at 30 min and 1 h. DNA fragmentation was lower in the control group compared to the treatment groups at 30 min (*p* < 0.05). However, at subsequent time points, DNA fragmentation was similar across all groups.

Regarding oxidative stress markers, there were trends indicating increased superoxide (Figure 2d,e; *p* > 0.05) and hydrogen peroxide (Figure 2f,g; *p* > 0.05) production with higher urea concentrations; however, these differences were not statistically significant. Over time, no significant changes were observed for any of the measured parameters (*p* > 0.05), except for live hydrogen peroxide-negative cells, which showed a significant increase (*p* = 0.005). Similarly, the treatment × time interaction was not significant for any parameter (*p* > 0.05).

## 4. Discussion

This study analyzed the impact of elevated ammonia and urea concentrations on frozen–thawed bovine sperm characteristics. The ammonia and urea concentrations were similar to those observed in uterine fluid levels in a previous study [10] investigating the impact of diets designed to induce low and high PUN concentrations. Although sperm motility is a critical parameter for assessing sperm quality [24], motility and kinematic parameters alone cannot fully predict fertilizing capacity. Sperm function within the intricate microenvironments of the female reproductive tract is paramount [25]. Numerous factors, including the presence of specific compounds in female genital tract [23], can significantly influence sperm motility characteristics.

In this study, ammonia and urea were tested separately to allow clear interpretation of their individual effects at physiologically relevant concentrations. While combined exposure could potentially reveal additive or synergistic effects, this was not included due to practical constraints. Future studies should therefore investigate whether simultaneous exposure impacts sperm function in an additive or synergistic manner.

Furthermore, the semen samples used in this study were sourced from a single, healthy bull to ensure experimental consistency and minimize inter-ejaculate variability, a standard approach for initial mechanistic toxicological studies [26,27]. While this allows for a clear interpretation of the treatment effects, it may limit the direct extrapolation of the results to the broader bull population, as individual variation in susceptibility to toxicants is well-documented [28]. Future studies should validate these findings using semen from a larger cohort of bulls representing different breeds, ages, and physiological states to assess the full scope of this variation.

In previous studies, high levels of NH3 and/or urea in blood and the uterine environment, often observed in cattle fed high-nitrogen diets, were associated with reduced conception rates [5,7,9,29]. This suggests that these metabolites potentially have an effect on the oviduct–uterine environment. In the present study, we observed significant effects of ammonia on both sperm motility and MMP. Exposure to elevated ammonia concentrations resulted in a significant decrease in total and progressive motility, as well as VAP, VCL, and VSL. The observed reductions in all these motility parameters demonstrate a comprehensive negative impact of ammonia on sperm movement. Our finding that HA severely impairs bull sperm motility and mitochondrial function is consistent with trends observed in other species but reveals important species-specific sensitivities. For instance, Zhao et al. [30] reported that ammonia significantly reduced motility in porcine sperm, though the effective concentrations were higher (>5 mM) than those used in our bovine model. This discrepancy may be attributed to inherent differences in sperm metabolism and membrane composition between species.

A key finding of this study was the significant impairment MMP by HA, even in the absence of measurable oxidative stress [31]. This indicates a targeted and specific toxicological effect of ammonia on sperm mitochondria [32], which appear to be exceptionally sensitive to this metabolite. The consistently depressed MMP in the HA group strongly suggests a direct compromise of mitochondrial function, which would critically undermine ATP production [33] and, consequently, the energy-dependent processes of motility and capacitation [34]. This specific mechanism of mitochondrial toxicity aligns with and provides a plausible explanation for the severe reproductive outcomes observed in other models. For instance, our results are consistent with the work of Zhang et al. [35] in mice, which documented not only reduced sperm function but also inheritable reductions in fertility following exposure to ammonia, highlighting its profound and potentially long-lasting impact on mammalian gametes. Given that mitochondria are the primary sites of ATP production, which is essential for sperm motility and capacitation and, hence, fertilization [32,33], this impairment is likely to have significant functional consequences. This observation is consistent with Agnihotri et al. [34], who reported that reduced MMP is linked to diminished sperm energy production and decreased fertility potential.

One limitation of this study is that real-time measurement of the ammonia and urea concentrations during incubation and a complete concentration–response analysis were not performed. While enzymatic breakdown of urea or microbial contamination might potentially affect metabolite concentrations, this was avoided by the brief incubation duration (≤4 h), the inclusion of antibiotics in the extender, and the low urease activity anticipated for frozen–thawed semen. Furthermore, the concentrations used were based on physiological levels reported for boar reproductive fluids [10] and thus were biologically relevant even if small deviations existed. However, in the absence of dynamic measurements, complete distinction between the direct impact of urea and indirect effects mediated by ammonia cannot be made. Future investigations should therefore include real-time metabolite measurement and more extensive dose–response designs to further elucidate the mechanisms of urea- and ammonia-induced changes in sperm function.

A further limitation of the present study is the dose design. While urea concentrations were within reported physiological ranges, a complete gradient (low, medium, high) was not included, which restricts the power to detect optimal or threshold effects. Ammonia concentrations, by contrast, were 2–23 times above physiological limits, so the negative effects observed on sperm motility and mitochondrial function are likely to reflect toxic, supraphysiological conditions rather than normal physiological mechanisms. Nevertheless, these findings underscore the sensitivity of bovine sperm to increased nitrogen metabolites. A wider range of urea doses and ammonia gradients within physiological ranges should be used in future research in order to resolve dose–effect relationships and separate physiological from toxic effects.

Interestingly, despite the significant effect of HA on MMP, we did not observe a clear effect of HU on sperm motility and kinematic parameters. This contrasts with a recent study on Holstein-Friesian bulls by Lavanya et al. [13], which reported a significant decline in motility and viability at a similar concentration (30 mM). A key methodological difference that may explain this divergence is the duration of exposure; Lavanya et al. [13] employed a 6 h incubation, whereas our assessment was conducted over 4 h. This suggests that the negative effects of urea on bull sperm may be time-dependent, manifesting more clearly in longer-term exposures. Furthermore, it underscores that mitochondrial dysfunction (observed in both studies) is likely an early event preceding the outright loss of motility. This suggests that while both ammonia and urea may have some impact on sperm function, their mechanisms of action may differ. This is supported by the observation that HA concentrations had a more pronounced and immediate effect on sperm viability, suggesting distinct pathways of toxicity. This heightened sensitivity to ammonia is consistent with findings in other species, including boars [30] and mice [35], where HA concentrations have been shown to be detrimental to spermatozoa. Although both compounds may influence various sperm parameters, ammonia’s negative impact on MMP and motility was the most prominent and statistically significant finding in this study.

Regarding oxidative stress markers, this study did not reveal a significant effect of HU or ammonia levels on superoxide or hydrogen peroxide production. This contrasts with numerous studies reporting an impact of these metabolites on oxidative stress in various cell types and biological systems [36,37]. For example, studies in hepatocytes indicated that ammonia induces oxidative stress by disrupting mitochondrial function and increasing ROS production [37]. Similarly, elevated urea levels have been linked to oxidative damage in renal tissues [36]. The discrepancy between these findings and our results may be attributable to several factors specific to our experimental design. The concentrations of ammonia (1562 µM) and urea (26.9 mg/dL) used in this study, while relevant to physiological conditions associated with high-nitrogen diets in cattle [10], may not have been sufficiently high, or the exposure time (2 h) long enough, to induce significant oxidative stress in sperm under our in vitro conditions. Spermatozoa possess endogenous antioxidant defense mechanisms [37] that may have effectively counteracted the pro-oxidant effects of the tested concentrations. Furthermore, the composition of sperm extender, which may contain antioxidants, could have also contributed to mitigating oxidative stress. Consequently, while ROS might have been generated, they were likely effectively neutralized, preventing a net increase in detectable levels. Furthermore, it is possible that the primary mechanisms of ammonia and urea toxicity in sperm are not primarily mediated through ROS generation in the short term but rather through direct ionic or metabolic disruption, as suggested by the significant mitochondrial dysfunction we observed. This highlights that the absence of a net increase in ROS does not preclude other forms of cellular damage. Future studies should incorporate measurements of direct oxidative damage markers, such as malondialdehyde (MDA) for lipid peroxidation and protein carbonyl content for protein oxidation, to more fully evaluate the potential stealthy oxidative insult that may not be captured by ROS probes alone [31].

In the present study, the temporary decrease in DFI at 30 min in the urea-treated groups compared to the control was not maintained at subsequent timepoints. The biological relevance of this short-term alteration is uncertain. Urea has also been shown to impact sperm chromatin stability and DNA integrity in cattle as well as other animals, although the majority of studies indicate harmful effects on supra-physiological or chronic exposure conditions [12,13]. Since our observed decrease was temporary and not succeeded by sustaining differences at 1 or 2 h, it might result from natural variability or transient responses of chromatin structure immediately after thawing. Previous papers also suggest that, in certain conditions, spermatozoa may possess restricted DNA repair or chromatin remodeling activity [38], but there is no direct evidence for such mechanisms in frozen–thawed cattle sperm yet. Therefore, we interpret this finding cautiously and observe the need for future research with more DNA damage and repair markers to determine the role of transient changes in DFI under urea treatment.

## 5. Conclusions

These findings demonstrate that ammonia, more so than urea, can significantly impair sperm motility, mitochondrial function, and viability. These findings highlight the need for strategies to manage ammonia exposure in bovine reproductive environments, especially in animals consuming high-nitrogen diets. Nevertheless, these conclusions should be interpreted in light of certain limitations. The use of a limited range of metabolite concentrations restricts detailed dose–response interpretation; combined ammonia–urea treatments were not assessed; and direct measurements of oxidative damage markers, such as lipid peroxidation and protein oxidation, were not performed. Future studies addressing these limitations will be essential to validate and extend the present findings.

## Figures and Tables

**Table 1:** pH values of the prepared media.

Treatment	pH
**Control**	7.60 ± 0.14
**HA**	7.82 ± 0.1
**LA**	7.78 ± 0.06
**HU**	7.80 ± 0.07
**LU**	7.73 ± 0.01

**Table 2:** Least square means ± SEM of bull sperm motility descriptors after 0, 1, 2, and 4 h of in vitro incubation with varying concentrations of ammonia.

Sperm Motility Descriptors ^1^	Class ^2^	Time				SE ^3^	*p*-Value		
		0	1 h	2 h	4 h		Treatment	Time	T × T ^4^
**% Mot**	**Control**	34.5 ^a^	41.4	52.9 ^a^	41.2	5.56	0.008	0.929	0.199
	**LA**	46.5 ^ab^	36.0	35.1 ^b^	35.1				
	**HA**	29.1 ^ac^	30.4	27.6 ^b^	32.0				
**% Prog Mot**	**Control**	34.2 ^a^	49.8 ^a^	52.3 ^a^	39.5	4.97	0.0002	0.721	0.035
	**LA**	45.8 ^a^	35.5 ^b^	34.7 ^b^	34.6				
	**HA**	28.4 ^b^	29.9 ^b^	27.1 ^b^	30.9				
**VAP (µm/s)**	**Control**	43.5 ^a^	64.5 ^a^	59.9 ^a^	35.1	6.22	0.0001	0.002	0.010
	**LA**	60.4 ^b^	41.3 ^b^	38.0 ^b^	30.4				
	**HA**	37.2 ^a^	36.6 ^b^	19.2 ^c^	24.0				
**VCL (µm/s)**	**Control**	83.6 ^a^	125.7 ^a^	116.7 ^a^	66.8	12.22	0.0005	0.004	0.003
	**LA**	121.5 ^b^	74.1 ^b^	72.5 ^b^	64.2				
	**HA**	72.5 ^a^	74.8 ^b^	58.0 ^b^	54.9				
**VSL (µm/s)**	**Control**	37.1 ^a^	56.3 ^a^	49.8 ^a^	30.5	5.61	0.0001	0.001	0.024
	**LA**	51.1 ^b^	35.7 ^b^	32.0 ^b^	24.9				
	**HA**	31.4 ^a^	29.9 ^b^	24.0 ^b^	19.7				
**STR**	**Control**	0.84	0.86	0.83	0.85	0.02	0.612	0.618	0.398
	**LA**	0.79	0.86	0.83	0.81				
	**HA**	0.84	0.81	0.88	0.82				
**LIN**	**Control**	0.44	0.44	0.42	0.44	0.02	0.128	0.239	0.610
	**LA**	0.42	0.48	0.44	0.39				
	**HA**	0.43	0.40	0.40	0.36				
**WOB**	**Control**	0.52	0.53	0.51	0.51 ^a^	0.02	0.177	0.089	0.567
	**LA**	0.49	0.55	0.52	0.47 ^ab^				
	**HA**	0.51	0.49	0.50	0.43 ^b^				
**ALH (µm)**	**Control**	0.96 ^a^	1.37 ^a^	1.42 ^a^	0.82	0.13	0.0003	0.037	0.005
	**LA**	1.28 ^b^	0.88 ^b^	0.88 ^b^	0.85				
	**HA**	0.81 ^a^	0.87 ^b^	0.70 ^b^	0.71				
**BCF (Hz)**	**Control**	8.83 ^a^	9.61 ^a^	9.89 ^a^	8.60	0.97	0.0001	0.262	0.127
	**LA**	9.89 ^ab^	6.66 ^b^	6.97 ^b^	7.21				
	**HA**	6.92 ^ac^	6.29 ^b^	5.90 ^b^	6.71				

Different superscript letters (a, b, c) denote significant differences among treatments (*p* < 0.05). ^1^ Sperm motility parameters: % Mot (motile spermatozoa), % Prog Mot (progressive motility), VCL (curvilinear velocity), VSL (straight-line velocity), VAP (average path velocity), LIN (linearity), STR (straightness), WOB (wobble), ALH (amplateral head displacement), BCF (beat cross frequency). ^2^ Treatment groups: Control (C), Low Ammonia (LA), High Ammonia (HA). ^3^ Standard Error. ^4^ Interaction effects between treatment and incubation time.

**Table 3:** Least square means ± SEM of bull sperm motility descriptors after 0, 1, 2, and 4 h of in vitro incubation with varying concentrations of urea.

Sperm Motility Descriptors ^1^	Class ^2^	Time				SE ^3^	*p*-Value		
		0	1 h	2 h	4 h		Treatment	Time	T × T ^4^
**% Mot**	**Control**	34.5	41.4	52.9	41.2	6.70	0.378	0.494	0.507
	**LU**	35.9	33.5	42.6	36.9				
	**HU**	43.2	38.8	35.3	29.0				
**% Prog Mot**	**Control**	34.2	49.8 ^a^	52.3 ^a^	39.5	6.17	0.091	0.274	0.235
	**LU**	35.5	33.0 ^b^	42.2 ^ab^	35.9				
	**HU**	42.8	38.2 ^ab^	34.5 ^b^	27.8				
**VAP (µm/s)**	**Control**	43.5	64.5 ^a^	59.9 ^a^	35.1	7.67	0.083	0.001	0.241
	**LU**	48.0	41.6 ^b^	45.4 ^ab^	26.8				
	**HU**	54.3	51.2 ^ab^	36.5 ^b^	20.5				
**VCL (µm/s)**	**Control**	83.6	125.7 ^a^	116.7 ^a^	66.8	13.6	0.106	0.0008	0.154
	**LU**	95.9	83.8 ^b^	84.5 ^b^	57.2				
	**HU**	107.9	101.6 ^ab^	71.0 ^b^	43.7				
**VSL (µm/s)**	**Control**	37.1	56.3 ^a^	49.8	30.5	6.84	0.114	0.002	0.328
	**LU**	41.8	35.0 ^b^	38.8	22.5				
	**HU**	46.8	42.9 ^ab^	33.1	17.3				
**STR**	**Control**	0.84	0.86	0.83	0.85	0.02	0.986	0.966	0.874
	**LU**	0.85	0.84	0.85	0.83				
	**HU**	0.86	0.83	0.85	0.83				
**LIN**	**Control**	0.44	0.44	0.42	0.44	0.02	0.671	0.344	0.732
	**LU**	0.41	0.41	0.42	0.39				
	**HU**	0.43	0.42	0.46	0.38				
**WOB**	**Control**	0.52	0.53	0.51	0.51	0.02	0.385	0.114	0.690
	**LU**	0.49	0.49	0.53	0.47				
	**HU**	0.50	0.50	0.54	0.46				
**ALH (µm)**	**Control**	0.96	1.37 ^a^	1.42 ^a^	0.87	0.15	0.065	0.002	0.138
	**LU**	1.05	0.94 ^b^	1.06 ^ab^	0.75				
	**HU**	1.17	1.11 a^b^	0.87 ^b^	0.58				
**BCF (Hz)**	**Control**	8.83	9.61	9.89 ^a^	8.60	1.14	0.022	0.419	0.378
	**LU**	8.43	7.25	8.09 ^ab^	8.05				
	**HU**	9.20	7.69	6.57 ^b^	6.07				

Different superscript letters (a, b) denote significant differences among treatments (*p* < 0.05). ^1^ Sperm motility parameters: % Mot (motile spermatozoa), % Prog Mot (progressive motility), VCL (curvilinear velocity), VSL (straight-line velocity), VAP (average path velocity), LIN (linearity), STR (straightness), WOB (wobble), ALH (amplateral head displacement), BCF (beat cross frequency). ^2^ Treatment groups: Control (C), Low Ammonia (LA), High Ammonia (HA). ^3^ Standard Error. ^4^ Interaction effects between treatment and incubation time.

## Data Availability

The original contributions presented in this study are included in the article. Further inquiries can be directed to the corresponding author.
